# Effects of Jaw Periosteal Cells on Dendritic Cell Maturation

**DOI:** 10.3390/jcm7100312

**Published:** 2018-09-29

**Authors:** Jingtao Dai, Daniela Rottau, Franziska Kohler, Siegmar Reinert, Dorothea Alexander

**Affiliations:** Department of Oral and Maxillofacial Surgery, University Hospital Tübingen, Osianderstr 2-8, 72076 Tübingen, Germany; jingtao.dai@med.uni-tuebingen.de (J.D.); danir@gmx.de (D.R.); kohler-franzi@web.de (F.K.); siegmar.reinert@med.uni-tuebingen.de (S.R.)

**Keywords:** dendritic cell differentiation, jaw periosteal cells, osteogenic differentiation, co-culture

## Abstract

Clinical application of tissue engineering products requires the exclusion of immune responses after implantation. We used jaw periosteal cells (JPCs) as a suitable stem cell source and analyzed herein the effects of JPCs on dendritic cell maturation after co-culturing of both cell types. Peripheral blood mononuclear cells (PBMCs) were differentiated to dendritic cells (DCs) by the addition of differentiation cocktails for 7 days in co-culture with undifferentiated and osteogenically induced JPCs. The effects of JPCs on DC maturation were analyzed at the beginning (day 7), in the middle (day 14), and at the end (day 21) of the osteogenesis process. We detected significantly lower DC numbers after co-culturing with JPCs that have previously been left untreated or osteogenically differentiated for 7, 14, and 21 days. Using gene expression analyses, significantly lower IL-12p35 and -p40 and pro-inflammatory cytokine (IFN-γ and TNF-α) levels were detected, whereas IL-8 mRNA levels were significantly higher in DCs. Furthermore, osteogenic media conditions enhanced significantly IL-10 gene expression. We concluded that undifferentiated and osteogenically differentiated JPCs had an overall inhibiting influence on dendritic cell maturation. Further studies should clarify the underlaying mechanism in depth.

## 1. Introduction

In the clinical application of tissue engineering (TE) products, it is crucial to prevent an immune rejection of the in vitro developed constructs. Biomaterials used for the generation of TE products, could induce an immune reaction. Efforts should be undertaken to develop biomaterials with low immunogenic potential. Some strategies involve the integration of immunosuppressive factors within the core material of biomaterials. However, these attempts fail, due to temporary but not persistent protection. On the other site, the potential of mesenchymal stromal stem cells (MSCs) could help to create an immunosuppressive environment, thus avoiding an immune response.

For TE purposes, the use of autologous instead of allogeneic stem cell sources should be preferred. However, it has been proven repeatedly in the past that MSCs have the potential to inhibit or ameliorate immune responses after allogeneic administration, for instance, in graft-versus-host-disease [1,2]. This observation is based on the immunoregulatory properties of MSCs on players of the innate and adaptive immune system [3]. It has been shown that MSCs are capable to downregulate cyclin D2, and therefore arrest stimulated T cells at the G1 phase [4]. Additionally, MSCs from bone marrow significantly suppressed CD4^+^ and CD8^+^ T cell proliferation [5], whereby soluble factors secreted by them were probably involved in this phenomenon and not the induction of apoptosis [5].

It has been demonstrated that MSCs also exert their regulatory functions by inhibition of the complement system. MSCs bind through the receptors C3aR and C5aR the complement components C3 and C5, protecting them from apoptosis and promoting their proliferation [6]. Tu and co-authors detected constitutive expression of the complement inhibitor factor H by MSCs [7]. The depletion of factor H abolished the complement inhibitory capacities of MSCs and a pro-inflammatory environment containing interferon-γ and TNF-α enhanced production of factor H by MSCs.

The immunosuppressive properties of MSCs target not only T cells, but also antigen presenting cells. They inhibit, for instance, the differentiation of CD34^+^ hematopoietic progenitors or monocytes into mature dendritic cells [8]. DCs are key regulators bridging the innate and adaptive immune system. Mice lacking dendritic cells develop systemic autoimmunity [9]. Whereas, immature DCs possessing high endocytic activity and expressing low levels of HLA-DR and co-stimulatory factors, maintain self-tolerance, mature DCs trigger immune responses by T cell activation.

Since harvesting of bone marrow for the isolation of MSCs requires surgeries, which lead to chronic pain in some patients (bone marrow aspirates from iliac crest), the use of mesenchymal stem cells derived from periosteum harbors several advantages. The harvest procedure is minimally invasive leaving no chronic pain [10]. Periosteal tissue is predestinated for bone reconstruction therapies [11,12], since it is its natural function to form new bone tissue after injury and/or to remodel bone tissue continuously. Furthermore, it has been shown that the osteogenic capacity of periosteum tissue depends on the tissue source [13]. In this study, periosteal tissue from load-bearing areas, such as tibia and femur, showed a higher osteogenic capacity compared to that harvested from calvarial and rib bones. In the light of this experience and the high mechanical loading occurring within the jaw, we are convinced that jaw periosteal cells represent the most suitable stem cell source for clinical applications in oral and maxillofacial surgery.

Meanwhile, profound knowledge of the immunoregulatory properties of MSCs from bone marrow already exists. Even when it can be speculated that periosteal cells behave similarly concerning their immunomodulatory features, no investigations have been performed. Therefore, we analyzed in the present study for the first time the effects of untreated and osteogenically differentiated JPCs on monocyte-derived DC differentiation.

## 2. Materials and Methods

### 2.1. Isolation and Culture of JPCs

**Figure 1:**
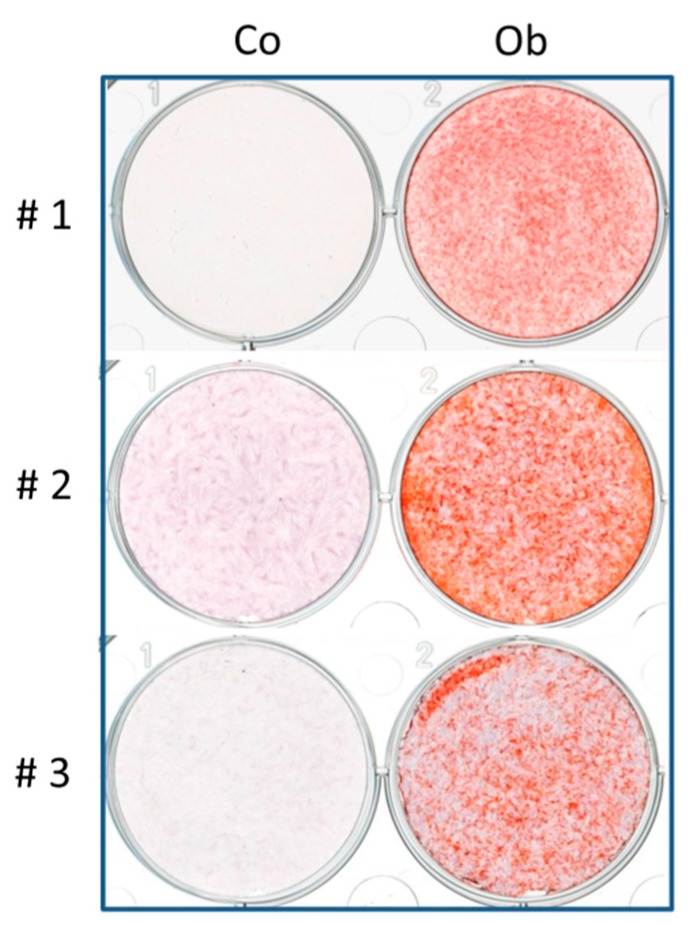
Alizarin staining of undifferentiated (co) and osteogenically differentiated (ob, 21 days) jaw periosteal cells (JPCs) derived from the 3 donors (#1, 2, 3) used for the following co-culture experiments.

The study was approved by the local ethics committee (Ethik-Kommission der Medizinischen Fakultaet Tuebingen; approval number 194/2008BO2). After obtaining written informed consent from all donors, the human jaw periosteum biopsy from 3 donors was obtained during routine interventions. The pieces (<1 cm^2^) of jaw periosteal tissue were cut in Dulbecco’s phosphate-buffered saline (DPBS w/o Mg^2+^, Ca^2+^, Sigma-Aldrich, Merck, Darmstadt, Germany) and enzymatically digested using 1500 U/mL type XI collagenase (Sigma-Aldrich, Merck, Darmstadt, Germany) for 90 min. The isolated JPCs were then plated in 75 cm^2^ cell culture flasks (Corning, Kaiserslautern, Germany) and cultivated under standard cell culture conditions (37 °C, 5% CO_2_, a humidified atmosphere of 95%). JPCs were routinely cultured in a 1:1 mixture of Dulbecco’s Modified Eagle Medium and Nutrient Mixture F-12 (Ham), containing GlutaMAX (DMEM/F-12, (Invitrogen-BioSource Europe, Thermo Fisher, Darmstadt, Germany) supplemented with 10% fetal bovine serum (FBS, Sigma-Aldrich, Merck, Darmstadt, Germany), 1% 250 μg/mL amphotericin B (Biochrom GmbH, Berlin, Germany), and 1% 10,000 U/mL penicillin/streptomycin (Lonza, Basel, Switzerland), for up to 4 passages. After reaching 80% confluence, JPCs were passaged with trypsin-versene EDTA (Lonza, Basel, Switzerland). Pooled JPCs from the three donors of passage 5 were used for all co-culture experiments, and cell medium was changed three times per week. The osteogenic potential of the used JPCs was tested and alizarin staining after 21 days of osteogenic differentiation is shown in Figure 1.

### 2.2. Isolation and Culture of PBMCs, Dendritic Cell Differentiation

Peripheral blood mononuclear cells (PBMCs) were collected and isolated from the blood of 6 normal healthy donors after informed consent. A whole blood sample was diluted 1:1 with DPBS and carefully layered over with 12 mL of 1.077 g/mL Ficoll-Paque PLUS (GE Healthcare, Freiburg, Germany). Plasma and red blood cells were separated from the PBMCs by density gradient centrifugation (no break, 810 g, 20 °C) for 20 min. The PBMCs were carefully harvested and transferred into another 50 mL tube. The cells were washed 3 times with DPBS, and then cultured in x-vivo 15 chemically defined, serum-free medium (Biozym, Hamburg Belgium) with 1% 10,000 U/mL penicillin/streptomycin and 3% autologous plasma. For flow cytometric analyses of dendritic cell marker expression, the PBMCs were grown in 75 cm^2^ culture flasks at a cell seeding density of 5 × 10^6^ cells per flask. For co-cultivation experiments, 6-well Transwell co-culture plates (0.4 μm pore size membrane, Corning, Kaiserslautern, Germany) with a cell seeding density of 1 × 10^6^ cells per well were used. To stimulate monocyte-derived dendritic cell differentiation, the first medium change was performed after 24 h, using x-vivo medium supplemented with 1% 10,000 U/mL penicillin/streptomycin, 3% autologous plasma, and the first DC differentiation cocktail containing 100 ng/mL GM-CSF (Sigma-Aldrich, Darmstadt, Germany) and 40 ng/mL IL-4 (Sigma-Aldrich, Darmstadt, Germany). At day 6, the second DC differentiation cocktail, containing 100 ng/mL GM-CSF, 40 ng/mL IL-4, 10 ng/mL TNF-α (Tebu Bio, Offenbach, Germany), 10 ng/mL IL-1β (Tebu Bio, Offenbach, Germany), 10 ng/mL IL-6 (Tebu Bio, Offenbach, Germany), and 1 µg/mL PGE2 (BioTrend, Köln, Germany) was added for another 24 h to stimulate DCs maturation. For co-culture experiments, pooled JPCs from 3 donors (upper chamber) and unpooled PBMCs from 6 donors were used in total. Co-cultures of PBMCs from one donor in the lower chamber with pooled JPCs from 3 donors (for all experiments the same donors) in the upper chamber were referred to as one independent experiment.

### 2.3. Flow Cytometric Analyses of Dendritic Marker Expression

PBMCs (derived from 4 donors) cultured under undifferentiated (day 1) and DC differentiation conditions (day 7) were collected from cell culture flasks. After centrifugation for 7 min (1400 rpm, 4 °C), cells were resuspended in 20 μL of 10% Gamunex (human immune globulin solution, Talecris Biotherapeutics, Frankfurt am Main, Germany) and incubated for 15 min at 4 °C. The cells were incubated at 4 °C with specific phycoerythrin (PE)-labeled mouse anti-human CD80, CD83, CCR-7 (BD Biosciences Pharmingen, Heidelberg, Germany) and HLA-DR (MACS Miltenyi Biotec, Bergisch Gladbach, Germany), and allophycocyanin (APC)-labeled mouse anti-human HLA-1, CD14 and CD86 (BioLegend, San Diego, CA, USA) for 15 min after adding 100 μL FACS buffer (DPBS, 0.1% sodium azide, 0.1% BSA). Subsequently, the cells were centrifuged for 7 min (1400 rpm, 4 °C) and washed two times with FACS buffer and then resuspended in 200 μL FACS buffer. The flow cytometric analyses were performed with the guava easyCyte 6HT-2L instrument (Merck Millipore, Germany). GuavaSoft 2.2.3 (InCyte 2.2.2) software was used for data evaluation.

### 2.4. Co-Cultivation of JPCs and PBMCs

The Transwell system was used to prevent JPCs from contacting PBMCs directly. Pooled JPCs (2 × 10^4^ in 1 mL of DMEM/F12) from 3 donors were seeded in the upper compartments of the 6-well Transwell co-culture plates. PBMCs (1 × 10^6^ per well in 2 mL of x-vivo medium) from 6 donors were cultured in the lower chambers (the ratio of JPCs to PBMCs was 1:50) in x-vivo medium with 1% 10,000 U/mL penicillin/streptomycin, 3% autologous plasma, and two DC differentiation cocktails (see above) for 7 days at 37 °C and 5% CO_2_, under four different upper chamber conditions:

1. JPC-free culture with complete DMEM/F12 medium (Monoculture_co);
2. JPC-free culture with osteogenic medium (ob—complete DMEM/F12 medium containing 100 mM L-ascorbic acid 2-phosphate, 10 mM β-glycerophosphate, and 4 µM dexamethasone, Sigma-Aldrich) (Monoculture_ob);
3. Co-culture with undifferentiated JPCs (Coculture_co);
4. Co-culture with osteogenic differentiated JPCs (Coculture_ob).

After 24 h of PBMCs (into the lower chamber) and JPCs (into the upper chamber) seeding in separate plates, a co-cultivation experiment was started, and the medium change in the upper chamber was performed on day 1 and day 3, respectively. To evaluate the effect of osteogenically differentiated JPCs on DCs, JPCs were either treated with osteogenic differentiation medium directly in the co-culture plates for day 7 of examination, or cells were induced in inserts from separate transwell plates for 7 and 14 days, before transferring the inserts to the co-culture plates with PBMCs/DCs for day 14 and 21 of examination. JPCs cultured without any osteogenic compounds for the same time period served as undifferentiated controls (co).

### 2.5. RNA Isolation and Quantitative Gene Expression Analyses in PBMCs

RNA isolation from PBMCs was performed with the NucleoSpin RNA XS kit (Macherey-Nagel, Hœrdt, France) following the manufacturer’s instructions. The isolated RNA was photometrically measured and quantified (GE Healthcare, Freiburg, Germany), and 200 ng of RNA were used for cDNA synthesis using the SuperScript VILO Kit (Invitrogen, Thermo Fisher, Darmstadt, Germany) following the manufacturer’s instructions. mRNA expression levels were quantified by the real-time LightCycler System (Roche Diagnostics, Mannheim Germany). DNA Master Sybr Green 1 (Roche, Mannheim, Germany) and commercial primer kits (Search LC, Heidelberg, Germany) were used for the PCR reactions. The following genes were analyzed: interleukin 12 subunit p35 (IL-12p35), interleukin 12 subunit p40 (IL-12p40), interleukin 12 receptor beta 1 (IL-12Rβ1), interleukin 12 receptor beta 2 (IL-12Rβ2), interferon-gamma (IFN-γ), tumor necrosis factor-alpha (TNF-α), interleukin 27 (IL-27), interleukin 8 (IL-8), and interleukin 10 (IL-10). Then, 40 cycles of PCR product amplification were carried out. The ratios of target genes versus the housekeeping gene glyceraldehyde 3-phosphate dehydrogenase (GAPDH, Search LC, Heidelberg, Germany) were calculated, and the ratios of PBMC monocultures (with normal (co) or osteogenic (ob) medium) were set as 1 (control) and induction factors (x-fold) in relation to this control were calculated.

### 2.6. IL-8 Protein Release in JPCs and PBMCs

The supernatants from JPCs and PBMCs were collected at day 1 and day 7, centrifuged (1400 rpm, 8 °C, 7 min), and kept at −80 °C before being assayed. IL-8 production in the medium was evaluated according to kit instructions (Invitrogen, Thermo Fisher, Darmstadt, Germany). All determinations were performed in duplicates. ELISA plates were read immediately with a Microplate ELISA reader (BioTek, Friedrichshall, Germany) at OD 450 nm. Concentrations of IL-8 were quantified with known standards, with the lowest detection limit of 15.63 pg/mL.

### 2.7. Statistical Analyses

All data were tested for normal distributions and variance equality. The results for surface marker expression were analyzed using the student’s *t*-test. Cell numbers and densities were analyzed by one-way analysis of variance (ANOVA) with culture type as an independent factor, whilst IL-8 concentrations were analyzed by two-way ANOVA with culture time and culture type as independent factors, followed by the Tukey HSD post-hoc test. The results of gene expression were analyzed using the Kruskal-Wallis test, followed by the Nemenyi post-hoc comparisons. Descriptive statistics were shown as the mean values ± standard error of mean of four (FACS analyses) or six independent experiments (co-culture experiments). Statistical analyses were performed using SPSS v.22.0 (IBM Corp., New York, NY, USA) at a level of significance of *p* < 0.05.

## 3. Results

### 3.1. Effect of DC Differentiation Cocktails on the Phenotype of Monocyte

**Figure 2:**
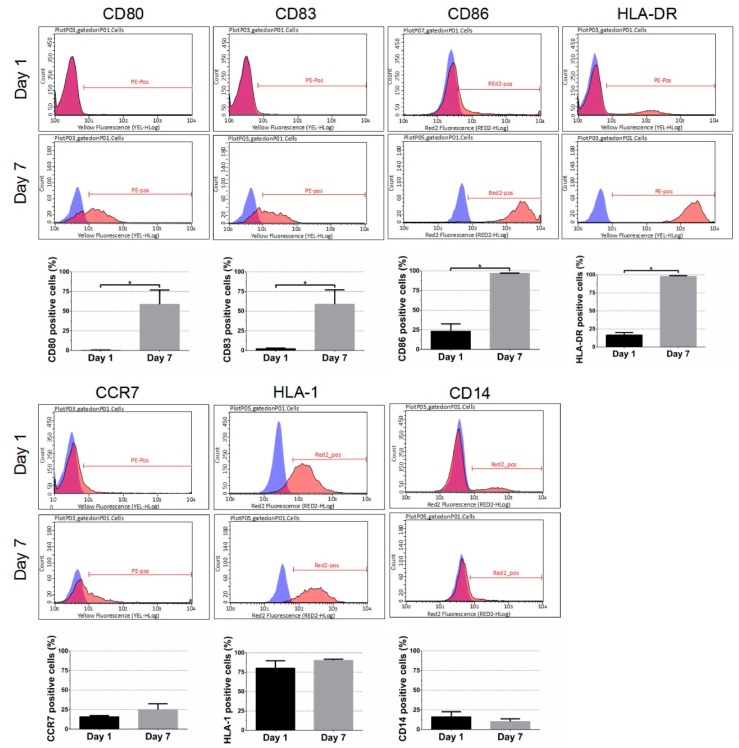
Flow cytometric analysis of cell surface marker expression before (day 1) and after DC differentiation (day 7). Representative flow cytometric histograms are illustrated for dendritic cell marker CD80, CD83, and CD86 expression. Furthermore, HLA-DR, CCR7, and the ubiquitous HLA-1 expression were analyzed. CD14 as a marker for mature monocytes is the only marker showing a decreasing tendency in expression. Results were averaged from 4 independent experiments.

To compare the phenotype of monocyte before and after DC differentiation, the cell surface marker expression was analyzed (from 4 donors). As shown in Figure 2, the expression of the costimulatory molecules CD80 and CD86, and of the DC maturation marker CD 83, and of the MHC II receptor HLA-DR were significantly up-regulated after 7 days of DC cultivation with the differentiation cocktails (CD80: day 7 58.71 ± 18.17% versus day 1 0.19 ± 0.41%, *p* < 0.05; CD86: day 7 96.78 ± 0.29% versus day 1 23.13 ± 9.42%, *p* < 0.05; CD83: day 7 58.82 ± 18.29% versus day 1 2.15 ± 0.94%, *p* < 0.05; HLA-DR: day 7 97.82 ± 1.04% versus day 1 16.71 ± 3.44%, *p* < 0.05). The difference in CD14 surface expression was not significant at day 7 compared to day 1 (day 7 10.24 ± 3.27% versus day 1 16.30 ± 6.36%, n.s.). These results indicated that monocyte-derived DCs develop the typical expression profile after stimulation with both DC differentiation cocktails, providing a basis for the further co-cultivation experiments.

### 3.2. Effect of JPCs on the Morphology, Number, and Size of DC

**Figure 3:**
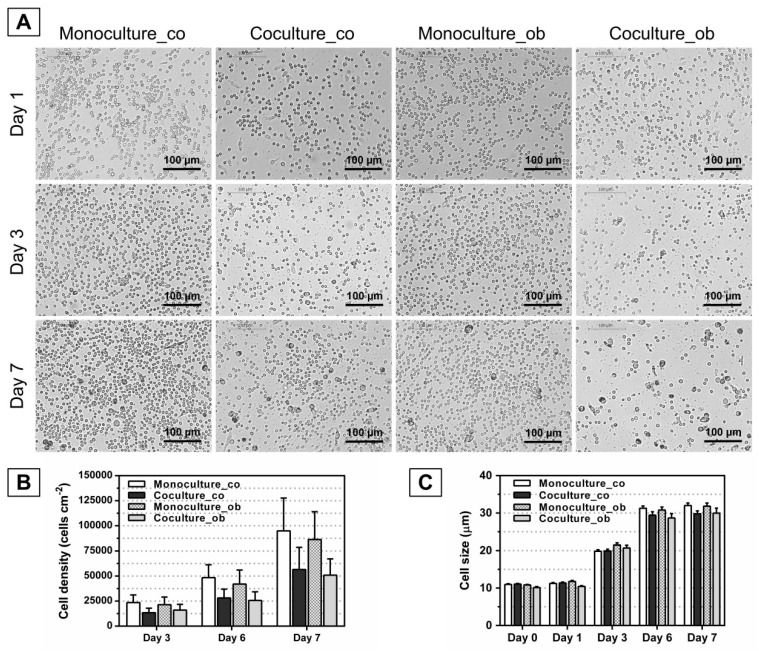
Morphology, cell numbers, and sizes of peripheral blood mononuclear cells (PBMCs) during DC differentiation in monocultures or co-cultures with untreated (co) and osteogenically induced JPCs for 7 days (ob). Microscopic images of PBMCs during DC differentiation on day 1, day 3, and 7 are shown (**part A**). Resulting cell numbers (cells/cm^2^, **part B**) and determination of cell sizes (µm, **part C**) in mono- and co-cultures were quantified using the ImageJ software. Results were averaged from 6 independent experiments. Scale bar, 100 µm.

**Figure 4:**
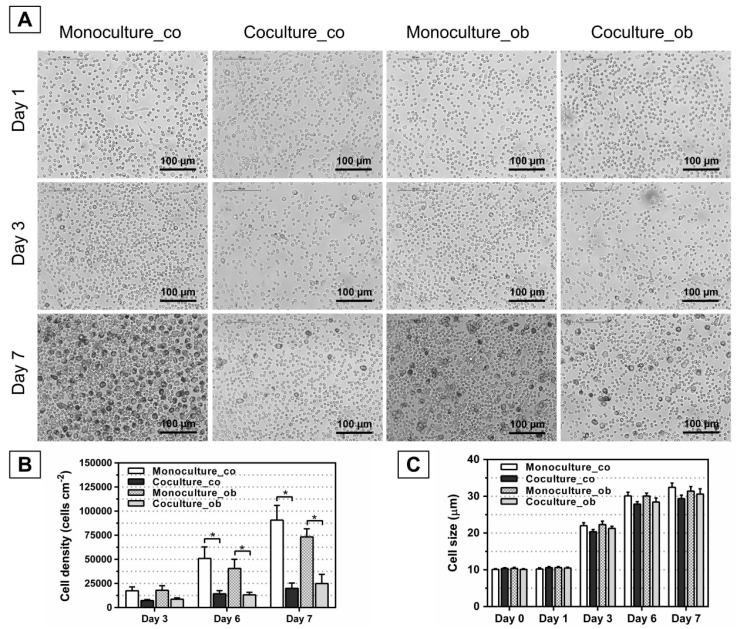
Morphology, cell numbers, and sizes of PBMCs during DC differentiation in monocultures or co-cultures with untreated (co) and osteogenically induced JPCs for 14 days (ob). Microscopic images of PBMCs during DC differentiation on day 1, day 3, and 7 are shown (**part A**); Resulting cell numbers (cells/cm^2^, **part B**) and determination of cell sizes (µm, **part C**) in mono- and co-cultures were quantified using the ImageJ software. Results were averaged from 6 independent experiments. Scale bar, 100 µm.

**Figure 5:**
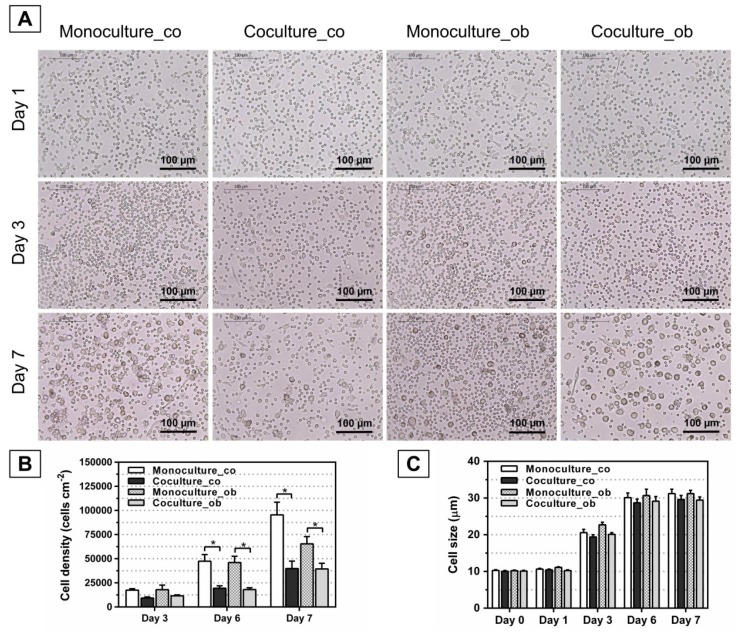
Morphology, cell numbers, and sizes of PBMCs during DC differentiation in monocultures or co-cultures with untreated (co) and osteogenically differentiated JPCs for 21 days (ob). Microscopic images of PBMCs during DC differentiation on day 1, day 3, and 7 are shown (**part A**); Resulting cell numbers (cells/cm^2^, **part B**) and determination of cell sizes (µm, **part C**) in mono- and co-cultures were quantified using the ImageJ software. Results were averaged from 6 independent experiments. Scale bar, 100 µm.

To investigate whether undifferentiated and osteogenically induced and/or differentiated JPCs inhibit the maturation of DCs, JPCs were cultured under normal (co) and osteogenic conditions (ob) for 7 days, 14 days, and 21 days, respectively. Light microscopy was used to identify DC, and the full-length cell sizes of mono- and co-cultured PBMCs from 6 donors were measured using ImageJ software (*n* = 30 per image). As shown in Figure 3A, Figure 4A, and Figure 5A, similar images were obtained, and the cells were found at day 1 as small round cells among all groups, whereas the cells were found to increase in size at day 7 compared to day 1; most importantly, less differentiated DCs were observed both in co-cultures with JPCs under untreated and osteogenic culture conditions. Furthermore, significantly reduced cell numbers were detected in co-cultures with JPCs at day 6 (Figure 4B co: co-culture 14,286 ± 3266 versus monoculture 50,947 ± 11,966, *p* < 0.05; Figure 3B ob: co-culture 13,236 ± 2629 versus monoculture 40,579 ± 9482, *p* < 0.05; Figure 5B co: co-culture 19,380 ± 2577 versus monoculture 47,491 ± 6801, *p* < 0.05; Figure 5B ob: co-culture 18,100 ± 1809 versus monoculture 46,109 ± 6461, *p* < 0.05) and day 7 (Figure 4B co: co-culture 19,995 ± 5389 versus monoculture 90,707 ± 15,196, *p* < 0.05; Figure 4B ob: co-culture 24,987 ± 9389 versus monoculture 73,272 ± 8384, *p* < 0.05; Figure 5B co: co-culture 39,785 ± 7803 versus monoculture 95,469 ± 13,045, *p* < 0.05; Figure 5B ob: co-culture 39,452 ± 5838 versus monoculture 65,489 ± 7465, *p* < 0.05). Whereas results from Figure 3B showed no statistically significant differences, co-cultures with JPCs still showed a lower cell density compared to monocultures (day 6 co: co-culture 28,198 ± 8753 versus monoculture 48,490 ± 12,841, n.s.; day 6 ob: co-culture 25,638 ± 8673 versus monoculture 42,034 ± 14,027, n.s.; day 7 co: coculture 56,580 ± 22,120 versus monoculture 94,931 ± 32,730, n.s.; day 7 ob: co-culture 50,896 ± 16,351 versus monoculture 86,354 ± 27,696, n.s.). In addition, as illustrated in Figure 3C, Figure 4C, and Figure 5C, the same tendency was observed, the cells had a diameter of 10 µm at day 0 and day 1. After treatment of monocytes with DC differentiation cocktails, an increased diameter (day 3: 20 µm; day 6 and day 7: 30 µm) was detected. However, the cell sizes were similar among all groups at the same time point of examination.

### 3.3. Effect of JPCs on DC Gene Expression

**Figure 6:**
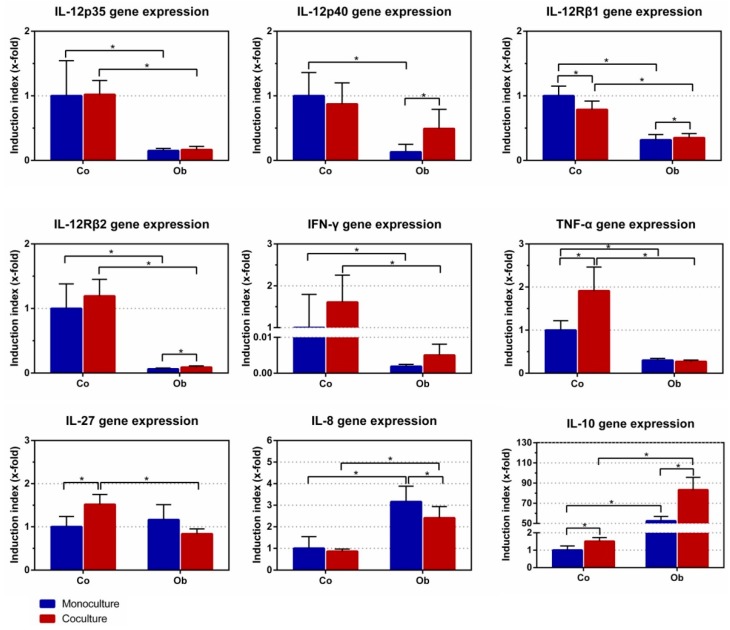
Quantitative gene expression in DCs (day 7 of differentiation) cultivated as monocultures or co-cultures with undifferentiated (co) and osteogenically induced JPCs for 21 days (ob). IL-12p35, IL-12p40, IL-12Rß1, IL-12Rß2, IFN-γ, TNF-a, IL-27, IL-8, and IL-10 gene expressions were quantified by the Light Cycler system, and ratios of listed genes in relation to the housekeeping gene GAPDH were calculated. Gene levels in DC monocultures (with control (co) medium) were set as 1, and induction indices (x-fold) in relation to this control were calculated. Results were averaged from 6 independent experiments.

To evaluate the effect of JPCs on DC gene expression, monocytes were cultured with differentiation cocktails containing GM-CSF, IL-4, IL-6, IL-1β, TNF-α, and PGE2 for 7 days as monocultures together with control/osteogenic (co/ob) medium in the upper chamber, and as co-cultures with untreated/osteogenically induced JPCs in a transwell system. DCs cultivated in the presence of untreated JPCs showed significant down-regulation of IL-12Rβ1 (0.79 ± 0.13-fold, *p* < 0.05) and up-regulation of three genes (TNF-α: 1.91 ± 0.55-fold, *p* < 0.05; IL-27: 1.52 ± 0.23-fold, *p* < 0.05; IL-10: 1.51 ± 0.22-fold, *p* < 0.05), compared to DC monocultures with control medium (set as 1) (Figure 6). However, DCs cultivated in the presence of osteogenically induced JPCs showed significant down-regulation of IL-8 (0.84 ± 0.12-fold, *p* < 0.05) and up-regulation of four genes (IL-12p40: 24.09 ± 6.57-fold, *p* < 0.05; IL-12Rβ1: 1.38 ± 0.34-fold, *p* < 0.05; IL-12Rβ2: 1.33 ± 0.18-fold, *p* < 0.05; IL-10: 1.68 ± 0.32-fold, *p* < 0.05), compared to DC monocultures with osteogenic (ob) medium. After exposure to osteogenic (ob) medium, gene expression by DCs showed a different pattern. This was further demonstrated by comparing DCs cultured with control/osteogenic (co/ob) medium and untreated or osteogenically induced JPCs, which showed statistically significant differences in the gene expression levels (Figure 6). As shown in the figure, DC monocultures cultivated in the presence of osteogenic (ob) medium showed significant down-regulation of six genes (IL-12p35: 0.15 ± 0.04-fold, *p* < 0.05; IL-12p40: 0.13 ± 0.12-fold, *p* < 0.05; IL-12Rβ1: 0.32 ± 0.08-fold, *p* < 0.05; IL-12Rβ2: 0.06 ± 0.01-fold, *p* < 0.05; IFN-γ: 0.0019 ± 0.0006-fold, *p* < 0.05; TNF-α: 0.30 ± 0.04-fold, *p* < 0.05) and significant up-regulation of two genes (IL-8: 3.16 ± 0.72-fold, *p* < 0.05; IL-10: 52.51 ± 4.48-fold, *p* < 0.05), compared to the control (co) medium (set as 1). Most importantly, co-cultures DCs cultivated in the presence of osteogenically induced JPCs showed significant down-regulation of six genes (IL-12p35: ob 0.17 ± 0.05-fold versus co 1.02 ± 0.22-fold, *p* < 0.05; IL-12Rβ1: ob 0.35 ± 0.07-fold versus co 0.79 ± 0.13-fold, *p* < 0.05; IL-12Rβ2: ob 0.09 ± 0.02-fold versus co 1.19 ± 0.26-fold, *p* < 0.05; IFN-γ: ob 0.005 ± 0.003-fold versus co 1.61 ± 0.65-fold, *p* < 0.05; TNF-α: ob 0.27 ± 0.03-fold versus co 1.91 ± 0.55-fold, *p* < 0.05; IL-27: ob 0.84 ± 0.12-fold versus co 1.52 ± 0.23-fold, *p* < 0.05) and significant up-regulation of two genes (IL-8: ob 2.41 ± 0.52-fold versus co 0.86 ± 0.11-fold, *p* < 0.05; IL-10: ob 83.30 ± 12.40-fold versus co 1.51 ± 0.22-fold, *p* < 0.05), compared to DC co-cultures with undifferentiated JPCs, as illustrated in Figure 6. These nine gene expression patterns showed a similar tendency in the groups of osteogenically differentiated JPCs for 7 days (as shown in Supplemental Figure S1) and for 14 days (as shown in Supplemental Figure S2).

### 3.4. Effect of JPCs on IL-8 Secretion of DC

**Figure 7:**
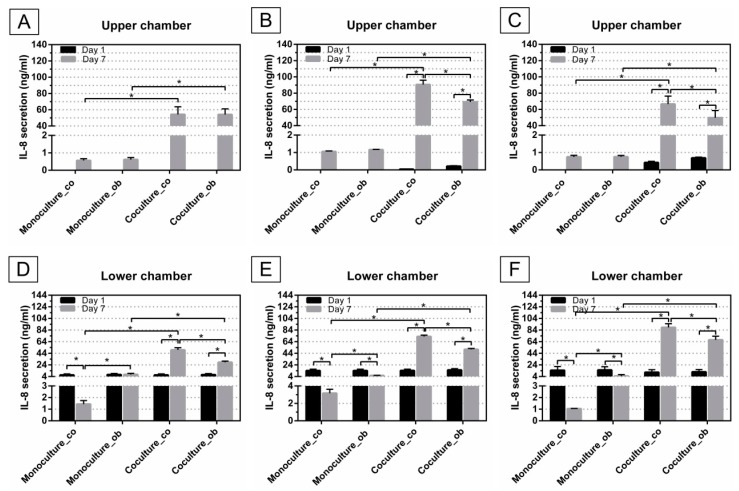
Analysis of IL-8 secretion in supernatants from upper and lower chamber of mono- and co-cultures, before and after DC differentiation (at day 1 and day 7). PBMCs/DCs were cultured in mono- and co-cultures with JPCs (untreated—co or osteogenically induced for 7 (**A**,**D**); 14 (**B**,**E**); and 21 (**C**,**F**) days) and IL-8 levels in supernatants from upper chambers of co-culture plates (upper panel) and lower chambers of co-culture plates (lower panel) were quantified by ELISA. Results were averaged from 6 independent experiments.

Monocytes were cultured with DC differentiation cocktails in the presence and absence of JPCs for 7 days, supernatants from these cells were collected for cytokine quantification via ELISA. The amounts of IL-8 secretion were analyzed, as illustrated in Figure 7. After culturing of JPCs for 14 days, the largest amounts of IL-8 were secreted by untreated JPCs (90.51 ± 5.39 ng/mL), while significantly lower levels of IL-8 were released by osteogenically differentiated JPCs (69.16 ± 2.46 ng/mL, *p* < 0.05) (Figure 7B). Levels of IL-8 released by JPCs cultured for 24 h were below the detection limit of the test, however, after co-cultures with PBMCs for 7 days, the IL-8 concentration in co-cultures was strongly increased (co: 54.22 ± 9.43 ng/mL; ob: 54.14 ± 6.88 ng/mL) (Figure 7A). The same tendency was observed in Figure 7B (co: day 7 90.51 ± 5.39 ng/mL versus day 1 0.04 ± 0.007ng/mL, *p* < 0.05; ob: day 7 69.16 ± 2.46 ng/mL versus day 1 0.21 ± 0.03 ng/mL, *p* < 0.05) and Figure 7C (co: day 7 66.54 ± 9.77 ng/mL versus day 1 0.42 ± 0.07 ng/mL, *p* < 0.05; ob: day 7 49.69 ± 8.94 ng/mL versus day 1 0.69 ± 0.05 ng/mL, *p* < 0.05), after co-cultures with PBMCs, the IL-8 expression levels were significantly higher. It should be noticed that after co-cultures with PBMCs, IL-8 production in osteogenically induced JPCs was significantly down-regulated compared to untreated JPCs in Figure 7B (ob 69.16 ± 2.46 ng/mL versus co 90.51 ± 5.39 ng/mL, *p* < 0.05) and Figure 7C (ob 49.69 ± 8.94 ng/mL versus co 66.54 ± 9.77 ng/mL, *p* < 0.05). However, this tendency was not observed in Figure 7A. In addition, after co-cultivation experiments, IL-8 secretion by untreated or osteogenically induced JPCs was significantly increased, compared to monocultures with control (co) or osteogenic (ob) medium in the upper chamber, respectively (Figure 7A–C).

Similarly, supernatants from the lower chamber were collected and analyzed using ELISA. IL-8 production was significantly decreased in monocultures with control (co) or osteogenic (ob) medium at day 7, compared to day 1 in Figure 7D (co: 1.43 ± 0.31 ng/mL versus 6.69 ± 1.84 ng/mL, *p* < 0.05), Figure 7E (co: 3.16 ± 0.46 ng/mL versus 14.22 ± 2.45 ng/mL, *p* < 0.05; ob: 5.76 ± 0.33 ng/mL versus 14.17 ± 2.48 ng/mL, *p* < 0.05), and Figure 7F (co: 1.04 ± 0.02 ng/mL versus 14.65 ± 6.48 ng/mL, *p* < 0.05; ob: 4.79 ± 2.47 ng/mL versus 15.23 ± 5.53 ng/mL, *p* < 0.05), with the exception from the monoculture_ob group in Figure 7D (7.55 ± 2.03 ng/mL versus 7.40 ± 1.95 ng/mL, n.s.). However, IL-8 concentration was significantly up-regulated in co-cultures with untreated (co) or osteogenically induced JPCs at day 7 compared to day 1, as shown in Figure 7D (co: 49.88 ± 3.75 ng/mL versus 6.64 ± 1.90 ng/mL, *p* < 0.05; ob: 28.58 ± 1.94 ng/mL versus 7.12 ± 1.93 ng/mL, *p* < 0.05), Figure 7E (co: 72.89 ± 2.32 ng/mL versus 14.37 ± 2.39 ng/mL, *p* < 0.05; ob: 50.76 ± 1.63 ng/mL versus 15.24 ± 2.29 ng/mL, *p* < 0.05) and Figure 7F (co: 88.64 ± 6.27 ng/mL versus 11.36 ± 4.49 ng/mL, *p* < 0.05; ob: 67.19 ± 6.27 ng/mL versus 11.97 ± 4.03 ng/mL, *p* < 0.05). Notably, at day 7, significantly higher amounts of IL-8 were secreted by monoculture DCs with osteogenic (ob) medium, compared to monoculture with control (co) medium in the upper chamber; whilst significantly lower amounts were detected in DCs co-cultures with osteogenically induced JPCs, compared to DCs co-cultures with undifferentiated JPCs, as shown in Figure 7D–F. In the presence of untreated or osteogenically induced JPCs, IL-8 secretion by DCs was significantly increased at day 7, compared to monoculture with control (co) or osteogenic medium, respectively (Figure 7D–F).

## 4. Discussion

In our study, we could demonstrate for the first time an immunosuppressive effect eliciting from jaw periosteal cells.

After the addition of IL-4 and GM-CSF at day 1, immature monocyte-derived DCs differentiate. The induction of terminal differentiated DCs requires the addition of the second cocktail at day 6, and at day 7 mature DCs were generated [14]. After stimulation, DCs upregulate surface expression of co-stimulatory factors CD80, CD83, and CD86, as well as expression of HLA-DR [15]. We could successfully demonstrate the generation of mature DCs by flow cytometric measurements of DC surface marker, as shown in Figure 2.

The quantification of dendritic cell densities during DC maturation (day 3, 6, and 7) resulted in significant decreases when DCs were co-cultured with JPCs, whereby higher differences were observed in the tendency in co-cultures with undifferentiated JPCs, as shown in Figure 3, Figure 4 and Figure 5 (day 6 and 7). However, significantly reduced dendritic cell numbers were counted likewise in co-cultures with osteogenically differentiated JPCs for 14 and 21 days, compared to monocultures cultivated with the respective media. Concerning the dendritic cell size, no significant differences were obtained; however, a tendency of diminished cell size in co-cultures with JPCs was evident. These results indicated clearly an inhibiting effect of JPCs on DC maturation, both in the undifferentiated and differentiated state.

Effects of JPCs on DC gene expression were much more complex. Figure 6 shows the overall effects of the osteogenic culture conditions on gene expression of DCs. Compared to monocultures with control medium in the upper chamber, addition of osteogenic medium led to overall lower gene expression of IL-12 p35, IL-12p40, the two subunits of the IL-12 receptor, as well as pro-inflammatory cytokine (IFN-γ and TNF-α) expression. On the other site, we detected significantly higher IL-8 and extremely high up-regulated IL-10 expression levels under osteogenic conditions. These results indicated an overall suppression of pro-inflammatory cytokines, except IL-8, and a strong induction of the anti-inflammatory cytokine IL-10 by osteogenic culture conditions. Compared to monocultures with osteogenic medium in the upper chamber, the effects emanating from osteogenically differentiated JPCs became evident. In these co-cultures, we detected significantly higher IL-12p40 expression levels and slightly higher IL-12Rβ1 and -β2 levels. At the same time, significantly lower IL-8 and significantly higher IL-10 expression levels were measured in co-cultures with osteogenically differentiated JPCs (for 21 days), in comparison to the respective monocultures. Although it might seem surprising at first sight because interleukin-10 is a physiologically relevant inhibitor of IL-12 secretion [16], it should be taken into consideration that we analyzed gene expression of the subunits p40 and p35. Natural killer stimulatory factor or interleukin 12 represents a major player in triggering T-helper 1 (Th1) responses [17,18]. It is a 70 kD heterodimeric pro-inflammatory cytokine composed of two covalently linked chains, p35 and p40, and is produced by antigen presenting cells [19]. The main physiological producers for IL-12 are phagocytes (monocytes/macrophages and neutrophils) and dendritic cells [20].

In mice, it has been proposed that the so-called biologically inert p40 homodimer represents a natural inhibitor of IL-12 due to its similar affinity to the IL-12Rβ1 chain competing with the biologically active p70 heterodimer [21]. However, other studies report of biological functions of the mouse p40 homodimer [22]. Kalinski and co-authors could demonstrate that prostaglandin E2, with the known Th2-driving function, selectively enhanced IL-12p40 gene and protein expression in human TNF-α activated immature DCs, and suppressed IL-12p70 production [23,24]. These results suggest that the IL-12p40 homodimer can inhibit IL-12p70 secretion, and consequently signal transduction. Unfortunately, we have not succeeded in measuring IL-12p70 protein levels in our co-cultures, limiting the examination of this hypothesis.

A recent study reports that MSCs secrete Galectin-1 and the authors postulate that this factor is able to inhibit dendritic cell function [25]. Furthermore, they detected increased Gal-1, IL-10, and IL-12 concentrations in supernatants from the co-culture system. Coinciding with the results from this study, we detected significantly higher IL-12p40 and IL-10 gene expression levels at the same time in DCs cultivated in co-cultures with osteogenically differentiated JPCs. We also detected elevated levels of Galectin-1 and -3 at the end of osteogenic differentiation of JPCs (unpublished data from our lab).

IL-27 is a member of the IL-12/IL-23 heterodimeric family of cytokines, capable of both enhancement or suppression of immune responses [26]. IL-27 induces expansion of Th1 and on the other hand, IL-27 suppresses immune responses through inhibition of the development of T helper 17 cells and induction of IL-10 production. In our study, osteogenically differentiated JPCs seemed to have no relevant influence on IL-27 gene expression.

Indoleamine 2,3-dioxygenase (IDO) and prostaglandin E2 (PGE2) are key regulators of MSC immunosuppressive activities. Meisel and co-authors demonstrated that MSCs can limit T cell responses via IDO-mediated tryptophan degradation [27]. Additionally, inhibitors of PGE2 synthesis mitigated the overall suppressive effects of MSCs [28]. We did not perform measurements of PGE2 levels in our co-cultures, since this hormone is a component of the DC differentiation cocktail. However, JPCs express high levels of IDO in both undifferentiated and osteogenically differentiated states (data not shown).

Mature type 1 polarizing DCs produce pro-inflammatory cytokines IL-1β, IL-12, IL-8, and TNF-α. Summarizing our results, we measured an overall induction of IL-8 gene expression (Figure 6) in mature DCs under osteogenic conditions, and also a significant reduction by co-culturing with osteogenically differentiated JPCs in comparison to the respective DC monocultures. The subsequent measurements of IL-8 protein levels showed very similar results: the osteogenic medium led to a significant upregulation of IL-8 release in DCs (Figure 7D–F monoculture_ob versus monoculture_co, day 7). Co-culturing with undifferentiated JPCs resulted in significant upregulation of IL-8 levels. We assume that this is probably the sum of IL-8 release from both cell types DCs and JPCs. However, we were not able to prove this hypothesis since JPC monoculture control was lacking in our experiments. IL-8 protein could also pass over the lower chamber into the upper chamber by diffusion. What is certain is that osteogenically differentiated JPCs were able to reduce significant maximal IL-8 levels achieved in co-cultures with undifferentiated JPCs. It seems on the one hand, that our used osteogenic medium triggers IL-8 release in mature DCs in contrast to findings from other publications concerning the effects of dexamethasone on different cell types [29,30,31]. On the other hand, significant reduction of IL-8 levels by osteogenically differentiated JPCs was remarkable, since IL-8 expression strongly increased during JPC osteogenesis (data not shown). The significantly reduced IL-8 levels in DCs at day 7 compared to that of PBMCs at day 1, could be because additional IL-8 producing cells were present in the cell suspension at this time point.

## 5. Conclusions

Taken together, our data revealed an overall immunosuppressive effect of undifferentiated and osteogenically induced and differentiated JPCs on monocyte-derived DC maturation. This finding is based on the detection of significantly lower numbers of differentiated DCs in the presence of inactivated or activated JPCs. The underlaying mechanism might involve overall repression of pro-inflammatory cytokines and high induction of the anti-inflammatory IL-10. Since this is the first study examining JPCs effects on immune cells, further studies should follow to clarify JPC mode of action more profoundly.

## Supplementary Materials

The following are available online at http://www.mdpi.com/2077-0383/7/10/312/s1, Supplemental results from gene expression analyses are illustrated in Figure S1: Quantitative gene expression in DCs (day 7 of differentiation) cultivated as monocultures or co-cultures with undifferentiated (co) and osteogenically induced JPCs for 7 days (ob). IL-12p35, IL-12p40, IL-12Rß1, IL-12Rß2, IFN-γ, TNF-a, IL-27, IL-8 and IL-10 gene expressions were quantified by the Light Cycler system and ratios of listed genes in relation to the housekeeping gene GAPDH were calculated. Gene levels in DC monocultures (with control (co) medium) were set as 1 and induction indices (x-fold) in relation to this control were calculated. Results were averaged from 6 independent experiments, Figure S2: Quantitative gene expression in DCs (day 7 of differentiation) cultivated as monocultures with osteogenic medium in the upper chamber or as co-cultures with osteogenically induced JPCs for 14 days (ob). The same genes as illustrated in Figure S1 were analyzed. Gene levels of DC monocultures (with JPC osteogenic (ob) medium) were set as 1 and induction indices (x-fold) in relation to this control were calculated. Results were averaged from 6 independent experiments.
