# Supplementary material for: Effects of Jaw Periosteal Cells on Dendritic Cell Maturation
Source: J Clin Med. 2018 Sep 29;7(10):312. doi: 10.3390/jcm7100312 (PMC6210277; doi:10.3390/jcm7100312)
Supplement: Supplementary file 1 [file jcm-07-00312-s001.pdf]

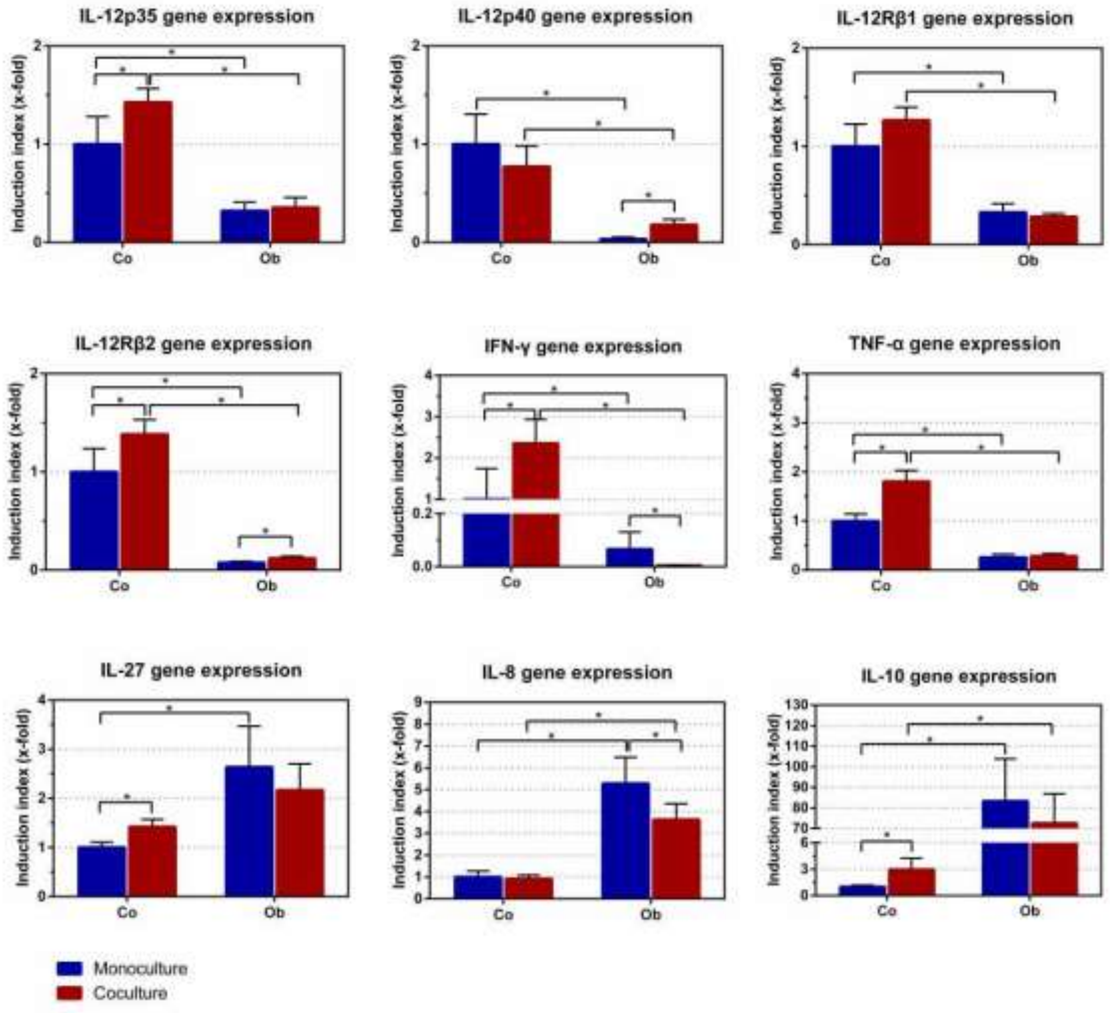

Figure S1. Quantitative gene expression in DCs (day 7 of differentiation) cultivated as monocultures or co-cultures with undifferentiated (co) and osteogenically induced JPCs for 7 days (ob). IL-12p35, IL-12p40, IL-12Rß1, IL-12Rß2, IFN-γ, TNF-a, IL-27, IL-8 and IL-10 gene expressions were quantified by the Light Cycler system and ratios of listed genes in relation to the housekeeping gene GAPDH were calculated. Gene levels in DC monocultures (with control (co) medium) were set as 1 and induction indices (x-fold) in relation to this control were calculated. Results were averaged from 6 independent experiments.

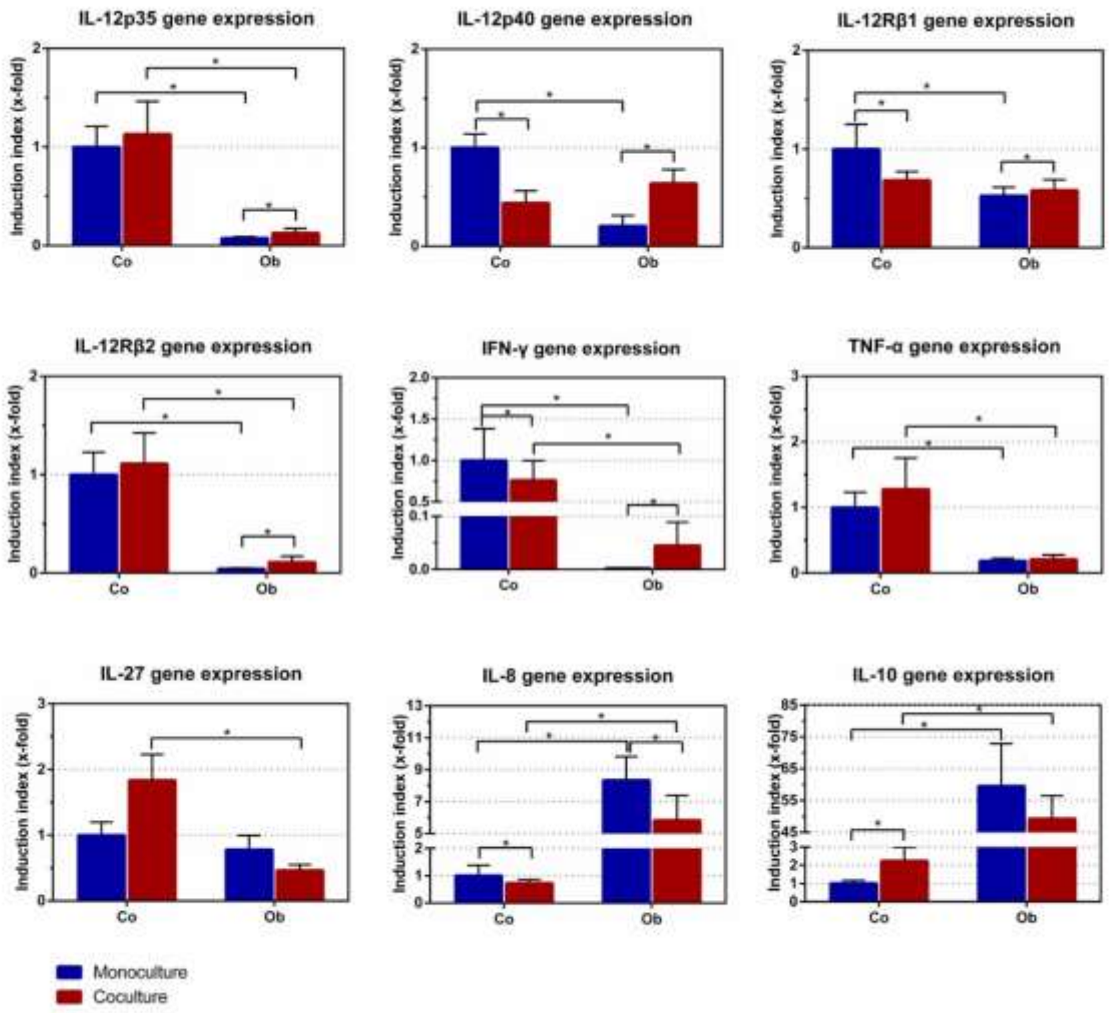

Figure S2. Quantitative gene expression in DCs (day 7 of differentiation) cultivated as monocultures with osteogenic medium in the upper chamber or as co-cultures with osteogenically induced JPCs for 14 days (ob). The same genes as illustrated in Figure S1 were analyzed. Gene levels of DC monocultures (with JPC osteogenic (ob) medium) were set as 1 and induction indices (x-fold) in relation to this control were calculated. Results were averaged from 6 independent experiments.
